# Gender-inclusive sexual health literacy scale for sex workers (SHL-SW): Development and validation in Thailand

**DOI:** 10.1371/journal.pone.0341345

**Published:** 2026-01-22

**Authors:** Saowanee Thongnopakun, Worarat Magteppong, Sawitree Visanuyothin, Jaturapon Charoenying, Orathai Thongnoppakun, Pitak Ketkrongkuay, Natsariya Chaksomsak, Aimutcha Wattanaburanon, Yuvadee Rodjarkpai, Tanunchai Boonnuk

**Affiliations:** 1 Faculty of Public Health, Burapha University, Chon Buri, Thailand; 2 Faculty of Nursing, Rajamangala University of Technology, Pathum Thani, Thailand; 3 National Health Security Office, Nakhon Ratchasima, Thailand; 4 Dusit Thani College, Chon Buri, Thailand; 5 Independent researcher, Chon Buri, Thailand,; 6 Pattaya Bhattamakun Hospital, Chon Buri, Thailand; 7 Faculty of Science and Technology, Loei Rajabhat University, Loei, Thailand; Commonwealth Scientific and Industrial Research Organisation, AUSTRALIA

## Abstract

**Background:**

Marginalized sex workers worldwide face persistent sexual health inequities, yet lack a validated, gender-inclusive tool to measure their sexual health literacy (SHL). This study aimed to develop and validate the Gender-inclusive Sexual Health Literacy Scale for Sex Workers (SHL-SW).

**Methods:**

A mixed methods approach was employed in three stages: (1) literature review, (2) expert consultation using the Delphi technique, and (3) questionnaire development and validation. The participants included 18 experts, 5 competent evaluators, and 600 sex workers. Data were collected through interviewer-administered online questionnaires. Analyses methods included median, interquartile range, content validity index, Cronbach’s alpha coefficient, and confirmatory factor analysis (CFA).

**Results:**

The final 10-item SHL-SW demonstrated high internal consistency (Cronbach’s α = 0.92). The CFA confirmed the hypothesized four-factor structure (access, understanding, appraisal, and application), with model fit indices indicating an excellent fit to the data (χ² = 20.568, p = 0.151; CFI = 0.99; TLI = 0.98; RMSEA = 0.04; SRMR = 0.03), thus establishing strong construct validity.

**Conclusion:**

The SHL-SW is a reliable and valid instrument for assessing SHL among sex workers in Thailand, and is the first such instrument to be specifically validated for gender inclusivity in this population across four components: (1) access, (2) understanding, (3) appraisal, and (4) application. This tool enables public health practitioners and researchers to identify SHL gaps, design targeted rights-based interventions, and inform evidence-based policies aimed at advancing health equity.

## Introduction

Sexually transmitted infections (STIs), including emergent threats (such as Mpox) and persistent challenges (such as gonorrhea and HIV/AIDS) constitute a significant global public health crisis [1,2]. The scale of the epidemic is alarming, with over one million new STI cases occurring daily worldwide [1]. This global challenge is keenly reflected in Thailand, where national surveillance data shows persistent high incidence rates for the five principal STIs, especially the upward trend in syphilis incidence (from 11 per 100,000 in 2018 to 28.1 in 2023) and the persistent HIV/AIDS epidemic [3,4].These statistics underscore the urgent need for effective, targeted prevention strategies in the country.

Sex workers are recognized as a key population in STI and HIV prevention strategies globally and in Thailand [5–7].In Thailand, surveillance data reveals concerning trends, including a steady decline in consistent condom use among sex workers [4].Moreover, epidemiological studies indicate significant disparities in health service engagement and health decision-making capacity within this group [8–10]. Notably, the sex worker population in Thailand is characterized by significant gender diversity [11], each group faces distinct social stigmas and health vulnerabilities, therefore they need different approach [12–14].

To address these complex challenges, Health Literacy (HL) has been identified as a critical strategy in global frameworks [15]. Consistent with the conceptual evolution from general health literacy frameworks [16], Sexual Health Literacy (SHL) is a specific subset of HL, defined as the social and cognitive skills that enable individuals to access, understand, appraise, and apply sexual health information to make informed decisions [17,18]. SHL is vital for empowering sex workers to navigate risks and negotiate safer sex [19]. Despite of this conceptual importance, a critical measurement gap exists, hindering the effective design of interventions. Systematic reviews confirm a scarcity of validated instruments designed specifically to measure SHL among sex workers [20,21]. Existing SHL tools were developed and validated in general populations, limiting their contextual relevance [22,23]. Critically, these existing tools are typically not designed to be gender-inclusive, thereby failing to capture the unique needs and experiences of a diverse sex worker population (e.g., transgender women and MSM) [19,20,24].This lack of a contextually and conceptually appropriate, gender-inclusive SHL measure constitutes the primary gap this research addresses.

Given this critical measurement gap and the context of Pattaya a global tourism hub where public health is intrinsically related to a safe tourism economy this study aimed to develop and validate the gender-inclusive Sexual Health Literacy Scale for Sex Workers (SHL-SW). The objective was to create a robust, contextually appropriate instrument to identify SHL needs and inform the design of targeted, rights-based interventions. Ultimately, this tool is intended to help reduce STI transmission, advance health equity for this marginalized population in Thailand, and serve as a foundational model for similar efforts worldwide.

## Materials and methods

### Study design

This study employed a mixed methods approach and was conducted from April 2024 to July 2024. The process was organized into two primary phases: (1) Literature review and construct definition, which involved literature reviews, item generation, and expert evaluation; and (2) item generation and content validation, where the scale was administered to a large sample and the measurement model’s goodness-of-fit was evaluated using confirmatory factor analysis (CFA).

#### The development of the gender-inclusive SHL-SW in Thailand.

The gender-inclusive SHL-SW was developed and validated in the following 3 steps:

**Step 1: Literature review and construct definition.** A comprehensive literature review was conducted to define SHL in the context of sex workers and to identify its key components. The proposed definition and domains were reviewed by 18 experts, including 6 policy experts, 6 academic experts, and 6 practitioners, who provided feedback on the appropriateness of the definition and the conceptual framework.

**Step 2: Item generation and content validation.** Using the Delphi technique, a preliminary pool of items was drafted based on expert input. The draft questionnaire was circulated to the same 18 experts for iterative review and refinement over 2 rounds. Content validity was assessed using the Content Validity Index (CVI). Additionally, five senior experts in measurement and evaluation—including (i) senior executives from the Ministry of Public Health; (ii) university professors specializing in health literacy, epidemiology, sexual health, and adult nursing; and (iii) senior executives from a leading NGO in family planning in Thailand—evaluated the item objective congruence (IOC). All items achieved an IOC score above the acceptable threshold of 0.50. In addition, reliability was preliminarily tested among 30 sex workers with similar characteristics to the main study participants.

**Step 3: Pilot testing and refinement.** The finalized 10-item questionnaire was administered to a sample of 600 sex workers of diverse genders. CFA was then performed on the collected data to evaluate the scale’s psychometric properties and construct validity.

#### Population, sample, and sampling technique.

The study participants were divided into four groups according to the purposes of instrument development and validation.

**Group 1: Experts for Delphi technique.** Eighteen experts were purposively recruited to participate in the Delphi process. The inclusion criteria required that all experts had worked in public health or health-related fields for at least three years and possessed relevant experience with regard to sex workers, such as sexually transmitted disease prevention and SHL. The expert panel comprised (i) six policy experts, including senior officials from national agencies (Department of Disease Control, Department of Health Service Support, Ministry of Public Health), regional agencies (Office of Disease Prevention and Control Region 6, Chonburi Provincial Public Health Office), and nongovernmental organizations (NGOs); (ii) six academic experts, who were university faculty members with expertise in health sciences and sexual behavior; and (iii) six practitioners, including local government health officers, district-level public health workers (e.g., community hospital staff), village health volunteers, and representatives from NGOs.

**Group 2: Experts for IOC.** Five senior experts evaluated the content validity of the instrument using the Index of Item Objective Congruence (IOC). The inclusion criteria for this group were at least five years of experience in evaluating the CVI and professional expertise in statistics, measurement and evaluation, or health sciences.

**Group 3: Pilot sample for reliability testing.** To preliminarily assess reliability, 30 sex workers of diverse genders with characteristics comparable to those included in the main study sample were recruited.

**Group 4: Main sample for CFA validation.** The main sample for the instrument’s psychometric validation consisted of 600 sex workers, stratified by sex assigned at birth into 300 females and 300 males. Participants were recruited in Pattaya City using a stratified two-stage cluster sampling method. First, the city was stratified into four geographical clusters: North Pattaya–Naklua Road, Central Pattaya Road, South Pattaya Road, and Thepprasit–Najomtien Road. Within each cluster, participants were recruited using accidental sampling, based on the willingness and cooperation of establishments and sex workers. Further, 150 respondents were obtained from each area to ensure both geographic coverage and gender balance.

Respondents considered eligible for participation were Thai nationals aged 18 years or older, who had worked as sex workers in Pattaya for at least three months; were mentally competent; able to read, speak, and communicate in Thai; and voluntarily provided informed consent. Data collection was conducted through structured online questionnaires administered via interviews.

### Data collection and data analysis

#### Data collection.

In the first step, an online questionnaire was developed to elicit expert opinions on the definition and components of the SHL-SW. The questionnaire consisted of two parts: (i) agreement or disagreement with the definition of SHL-SW proposed by the researchers, and (ii) agreement or disagreement with the proposed components of SHL-SW. Experts who disagreed were asked to provide their own definitions or alternative components. Descriptive statistics were employed to summarize expert feedback.

In the second step, the results from the first round were used to construct a framework for the questionnaire, which included 10 draft closed-ended items. Content validity was examined using the CVI. In this round, the revised questionnaire was distributed online to the 18 experts, who rated the relevance of each item using a five-point Likert scale (1 = Strongly Disagree, 2 = Disagree, 3 = Neutral, 4 = Agree, 5 = Strongly Agree). The proportion of expert ratings in the range of 4–5 was calculated for each item. Items with proportions below 0.83 were revised. At the questionnaire level, a minimum CVI threshold of 0.80 was applied. Experts’ ratings were further analyzed using the median and interquartile range (IQR). A median score of ≥4.50 indicated the highest relevance, while a score of 3.50–4.49 indicated high relevance. Items with an IQR not exceeding 1.5 were considered to have consensus among experts. The improved version of the questionnaire was circulated for a third round of expert review following the same CVI assessment process. In instances where comments were unclear, the researchers directly contacted experts for clarification before revising the items.

In the third step, construct validity was examined. The correlation matrix of the items was tested for factorability using Bartlett’s test of sphericity (χ² = 2390.901, df = 120, p < 0.001), which confirmed that the correlation matrix was significantly different from an identity matrix. Sampling adequacy was assessed using the Kaiser–Meyer–Olkin (KMO) measure, which was 0.913, thereby indicating that the data were suitable for factor analysis. Further, the measure of sampling adequacy (MSA) for individual items ranged from 0.885 to 0.965, all exceeding the acceptable threshold of 0.50, thereby further confirming appropriateness for structural analysis.

#### Data analysis.

CFA was conducted using LISREL 8.80 to test the hypothesized factor structure of SHL-SW. Since the observed variables met the assumption of multivariate normality and the data were independently distributed, without extreme skewness or kurtosis, maximum likelihood estimation (ML) was used for parameter estimation. The model fit was evaluated using multiple indices, including chi-square statistics, a goodness-of-fit index (GFI), the Adjusted Goodness-of-Fit Index (AGFI), the root mean square residual (RMR), the root mean square error of approximation (RMSEA), and the Akaike information criterion (AIC). Model modifications were considered based on the maximum of Modification Index (MI) ≥ 3.84 [25].

The final version of the online questionnaire consisted of 10 items and was administered for psychometric validation to the main study sample of 600 sex workers of diverse genders.

### Ethical considerations

Ethical approval for this study was obtained from Burapha University in accordance with the principles of the Declaration of Helsinki (Certification Code: HS001/2568(C1)). All participants provided written informed consent prior to participation and were fully informed regarding the study objectives and procedures; moreover, they were assured of confidentiality and protection of their privacy.

## Results

### Step 1: Definition and components of SHL-SW

Eighteen experts participated in the Delphi process to refine the definition and identify the components of the SHL-SW. All experts reached a consensus on the appropriateness of the proposed definition and its alignment with the context of sex workers in Thailand. The final following is the agreed-upon definition of SHL-SW:

*“Cognitive and social skills that play a crucial role in determining the motivation and potential of sex workers to access sexual health information and services for the prevention of sexually transmitted diseases (STDs); to understand information sufficiently for STD prevention; to appraise sexual health for STD prevention; and to make decisions and adopt sexual practices for the prevention of STDs.”*

Further, based on expert consensus, the following four key components of SHL-SW were established:

1. **Access:** Access to sexual health information and services for the prevention of STDs.
2. **Understanding:** Understanding of necessary information for the prevention of STDs.
3. **Appraisal:** Appraisal of sexual health for the prevention of STDs.
4. **Application:** Application of health decisions and practices for the prevention of STDs.

While the experts confirmed the theoretical soundness of the proposed definition and components, several experts emphasized the importance of linguistic clarity and cultural appropriateness. They recommended adapting the wording to ensure comprehensibility for sex workers across diverse educational and sociocultural backgrounds. Accordingly, minor revisions were made to simplify technical terminology and to better align with the lived experiences of sex workers.

### Step 2: Results of the CVI analysis

The final SHL-SW consisted of 10 items developed from the conceptual framework of health literacy. The scale comprised four domains: (i) access to sexual health information and services (three items), (ii) understanding of information (three items), (iii) appraisal of health information (two items), and (iv) application of health information in decision-making (two items).

Further, the development process employed a three-round Delphi technique. In the first round, content validity was assessed using the CVI. Most items achieved CVI values ≥0.83; however, one item (Item 4) had a CVI of 0.78, which did not meet the minimum threshold. In addition, several items (Items 1, 4, 5, 6, 7, 8, 9, and 10) received expert comments that indicated lack of clarity. These items were revised according to expert suggestions before proceeding to the second round.

In the second round, the scale-level CVI (S-CVI) was 0.89. Further refinements were made to improve clarity in wording based on expert recommendations. After the third round, all items achieved CVI values above 0.83; the overall S-CVI increased to 0.94, thus indicating excellent content validity (Table 1).

**Table 1:** Results of content validity evaluation across the second and third rounds of expert reviews (n = 18).

Item	Round 2		Round 3		CVI	Consensus
	Mean (SD)	Median (IQR)	Mean (SD)	Median (IQR)		
**Access to sexual health information and services for the prevention of sexually transmitted diseases (STDs) (access)**						
Q1. I am able to find and access information regarding STD prevention from diverse and credible sources, such as health care professionals, reputable online platforms, or trusted organizations.	4.61 (0.61)	5.0 (1.0)	4.67 (0.59)	5.0 (1.0)	0.94	Very strong agreement
Q2. I verify the credibility of STD-related information and health services by checking the source’s author, organization, and publication date.	4.61 (0.61)	5.0 (1.0)	4.67 (0.59)	5.0 (1.0)	0.94	Very strong agreement
Q3. I verify the validity of information regarding STDs before I act on it.	4.56 (0.78)	5.0 (1.00)	4.67 (0.49)	5.0 (1.0)	1.00	Very strong agreement
**Understanding of information sufficient for the prevention of sexually transmitted diseases (STDs) (Understand)**						
Q4. I can fully and correctly understand the information I read or hear on STD prevention.	4.61 (0.61)	5.0 (1.0)	4.61 (0.61)	5.0 (1.0)	0.94	Very strong agreement
Q5. I have difficulty recalling information regarding STD prevention from different media sources, such as the internet, television, or print materials.***	4.22 (0.94)	4.5 (1.3)	4.44 (0.62)	4.5 (1.25)	0.94	Very strong agreement
Q6. I can correctly interpret the symbols and numbers on health reports about STDs (e.g., understanding my HIV test results).	4.39 (0.85)	5.0 (1.0)	4.50 (0.62)	5.0 (1.0)	0.94	Very strong agreement
**Appraisal of sexual health for the prevention of sexually transmitted diseases (STDs) (Appraise)**						
Q7. It is difficult for me to verify the accuracy of information regarding STDs.***	4.22 (0.88)	4.0 (1.0)	4.28 (0.75)	4.0 (1.0)	0.83	Very strong agreement
Q8. I feel comfortable asking health care providers or individuals associated with trusted organizations questions regarding STDs when I am unsure about something.	4.56 (0.62)	5.0 (1.0)	4.61 (0.50)	5.0 (1.0)	1.00	Very strong agreement
**Application of health decisions and practices for the prevention of sexually transmitted diseases (STDs) (Apply)**						
Q9. I apply self-management skills such as goal-setting and planning when deciding how to protect myself from STDs or HIV (e.g., I decide to always use condoms, plan how to obtain them, and ensure I stick to my plan).	4.39 (0.78)	5.0 (1.0)	4.44 (0.70)	5.0 (1.0)	0.89	Very strong agreement
Q10. I can act on evidence-based recommendations from experts to prevent STDs.	4.56 (0.70)	5.0 (1.0)	4.61 (0.61)	5.0 (1.0)	0.94	Very strong agreement

### Step 3: Results of first- and second-order confirmatory factor analysis

CFA was conducted on data from 600 sex workers. The correlation coefficients among all observed variables were statistically significant (p < 0.001), ranging from 0.113 to 0.743, thus indicating positive correlations at low to moderate levels.

For the first-order CFA, the following four latent constructs were examined:

#### Factor 1: Access to sexual health information and services (HL1–HL3).

All items were significantly correlated (p < 0.001), with correlation coefficients ranging from 0.655 to 0.743, thereby reflecting moderate to high positive correlations.

#### Factor 2: Understanding of sexual health information (HL4–HL6).

Significant correlations were observed among items (p < 0.001), with coefficients ranging from 0.170 to 0.585, thereby representing low to moderate positive correlations.

#### Factor 3: Appraisal of sexual health information (HL7–HL8).

The two items demonstrated a significant positive correlation (p < 0.001), with a coefficient of 0.408, thus indicating a moderate correlation.

#### Factor 4: Application of health decisions and practices (HL9–HL10).

Both items were significantly correlated (p < 0.001), with a coefficient of 0.651, thereby indicating a moderate positive correlation.

Sampling adequacy was assessed prior to CFA. The KMO measure was 0.861, which exceeded the recommended threshold of 0.60 and, thus, confirmed the suitability of the data for factor analysis and multivariate analysis. Further, Bartlett’s test of sphericity was statistically significant (p < 0.001), thus indicating that the correlation matrix was not an identity matrix. The measure of sampling adequacy (MSA) for individual items ranged from 0.665 to 0.925, all above the acceptable level of 0.50. These results confirmed that the observed variables were sufficiently correlated to justify the use of structural factor analysis (Table 2).

**Table 2:** Correlation coefficients among observed variables in the Sexual Health Literacy Scale for Sex Workers (SHL-SW) model.

	HL1	HL2	HL3	HL4	HL5	HL6	HL7	HL8	HL9	HL10
HL1	0.919	0.655**	0.707**	0.612**	0.201**	0.485**	0.259**	0.376**	0.385**	0.433**
HL2		0.894	0.743**	0.627**	0.269**	0.538**	0.269**	0.345**	0.399**	0.467**
HL3			0.865	0.701**	0.198**	0.531**	0.285**	0.331**	0.446**	0.480**
HL4				0.906	0.170**	0.585**	0.273**	0.396**	0.489**	0.573**
HL5					0.665	0.285**	0.502**	0.121**	0.113**	0.126**
HL6						0.925	0.335**	0.268**	0.415**	0.404**
HL7							0.705	0.408**	0.213**	0.180**
HL8								0.825	0.318**	0.323**
HL9									0.842	0.651**
HL10										0.842
Mean	4.42	4.31	4.26	4.34	3.56	3.88	3.80	4.42	4.48	4.45
S.D.	0.82	0.87	0.89	0.83	1.35	1.24	1.26	0.85	0.69	0.71
Sk	−1.462	−1.207	−1.201	−1.138	−0.573	−0.889	−0.843	−1.844	−1.277	−1.293
Ku	1.802	1.010	1.124	0.674	−0.867	−0.314	−0.277	3.927	1.742	2.157

Bartlett’s Test $x_{45}^{2}$=2820.444, p < 0.001, Kaiser-Meyer-Olkin Measure of sample adequacy (KMO)=0.861

Notes: **Values on the diagonal (in grey cells) represent the Measures of Sampling Adequacy (MSA). - Correlation is significant at the 0.01 level (2-tailed).

#### Results of the first-order CFA.

The first-order CFA was performed to evaluate the measurement model of the SHL-SW. The factor loadings (b), standard errors (SE), t-values, and coefficients of determination (R²) for the 10 observed indicators were examined. All factor loadings were positive, ranging from 0.518 to 0.817, with standard errors between 0.023 and 0.066. All loadings were statistically significant at the 0.01 level. The coefficients of determination (R²) ranged from 0.143 to 0.886, thereby indicating acceptable levels of explained variance for the observed variables.

**Factor 1: Access to sexual health information and services**: Factor loadings ranged from 0.793 to 1.000, with SE values between 0.023 and 0.024.

**Factor 2: Understanding of sexual health information**: Factor loadings ranged from 0.619 to 1.000, with SE values between 0.027 and 0.066.

**Factor 3: Appraisal of sexual health information**: The factor loading was 0.518, with an SE of 0.040.

**Factor 4: Application of health decisions and practices**: The factor loading was 0.703, with an SE of 0.030.

The assessment of construct validity demonstrated satisfactory results. Convergent validity was supported by the average variance extracted (AVE), with values of 0.799, 0.521, 0.585, and 0.786 for Factors 1–4, respectively, all exceeding the threshold of 0.50. The construct reliability (CR) values were 0.922, 0.747, 0.735, and 0.880, respectively, all of which are above the recommended cutoff of 0.70. These results indicated that the measurement model had good convergent validity and high reliability, and that the model adequately fit the empirical data (Table 3).

**Table 3:** Results of first- and second-order confirmatory factor analysis of the Sexual Health Literacy Scale for Sex Workers (SHL-SW) model after model modification.

Component	Observed indicators	b(SE)	Β	t	R^2^	AVE	CR
**First order confirmatory factor analysis**							
**(Access)**	**HL1**	0.793(0.024)	0.857	32.691**	0.734	0.799	0.922
	**HL2**	0.817(0.023)	0.881	35.057**	0.775		
	**HL3**	1.000 (-)	0.941	–	0.886		
**(Understand)**	**HL4**	0.619(0.027)	0.906	23.145**	0.821	0.521	0.747
	**HL5**	0.620(0.066)	0.379	9.362**	0.143		
	**HL6**	1.000 (-)	0.773	–	0.597		
**(Appraise)**	**HL7**	0.518(0.040)	0.660	13.032**	0.436	0.585	0.735
	**HL8**	1.000 (-)	0.857	–	0.735		
**(Apply)**	**HL9**	0.703(0.030)	0.849	23.711**	0.721	0.786	0.880
	**HL10**	1.000 (-)	0.923	–	0.852		
**Second-order confirmatory factor analysis**							
**SHL-SW**	**(Access)**	0.323(0.016)	0.933	20.327**	0.871	0.769	0.929
	**(Understand)**	0.317(0.018)	0.994	17.584**	0.987		
	**(Appraise)**	0.653(0.043)	0.753	15.230**	0.567		
	**(Apply)**	1.000 (-)	0.806	–	0.649		

Notes: **Significant at the 0.01 level. Standard errors (SE) and t-values are not reported because these were constrained parameters.

#### Results of the second-order CFA.

**Fig 1:**
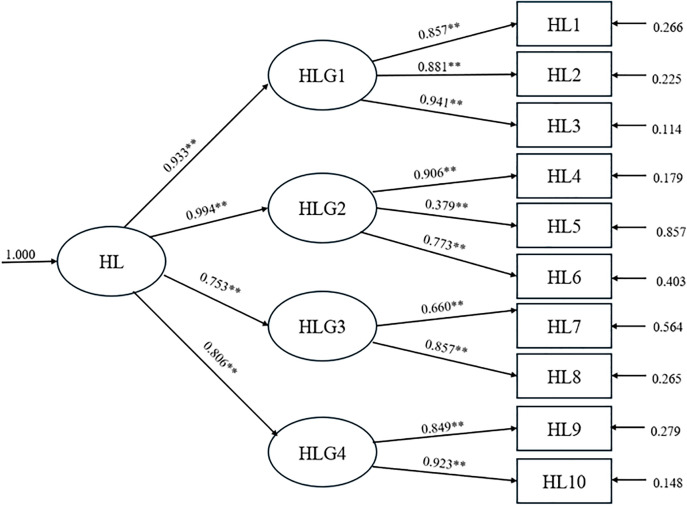
Confirmatory factor analysis (CFA) model of the Sexual Health Literacy Scale for Sex Workers (SHL-SW).

The second-order CFA was performed to examine the hierarchical structure of the SHL-SW. All factor loadings (b) of the four first-order constructs on the higher-order construct were statistically significant at the 0.01 level. Among the four domains, the application of health decisions and practices (application) had the highest factor loading (b = 1.00), followed by appraisal of sexual health information (appraisal) (b = 0.653), access to sexual health information and services (access) (b = 0.323), and understanding of sexual health information (understanding) (b = 0.317). Standard errors (SE) ranged from 0.016 to 0.043, while the coefficients of determination (R²) were 0.871, 0.987, 0.567, and 0.649, respectively. These findings indicated that all four domains were valid and reliable indicators of the higher-order construct of SHL among sex workers (Fig 1; Table 4).

**Table 4:** Goodness-of-fit and harmony indices of the Sexual Health Literacy Scale for Sex Workers (SHL-SW) model.

Goodness-of-fit and harmony indices	Evaluation criteria	Before model modification		After model modification	
		Value	Evaluation criteria	Value	Evaluation criteria
χ^2^ p-value	Not statistically significant (p > 0.05)	χ^2^ = 410.300, p < 0.0001	Not acceptable	χ^2^ = 20.568, p = 0.151	acceptable
χ^2^/df	<2.00	13.235	Not acceptable	1.371	acceptable
CFI	≥ 0.90	0.954	acceptable	0.999	acceptable
GFI	≥ 0.90	0.888	Not acceptable	0.993	acceptable
NFI	≥ 0.90	0.951	acceptable	0.998	acceptable
AGFI	≥ 0.90	0.802	Not acceptable	0.975	acceptable
SRMR	< 0.05	0.063	Not acceptable	0.015	acceptable
RMSEA	< 0.05	0.136	Not acceptable	0.025	acceptable

Note: Indices used to assess the goodness-of-fit of the model with the empirical data.

Further, the assessment of construct validity revealed good results. Convergent validity was supported by the average variance extracted (AVE = 0.769), which exceeded the recommended threshold of 0.50. Construct reliability (CR) was 0.929, thereby surpassing the acceptable cutoff of 0.70 and indicating strong reliability.

Model fit was evaluated before and after model respecification. Prior to modification, several fit indices did not meet recommended thresholds. After model modification based on modification indices and theoretical considerations, the model achieved an excellent fit with the empirical data: χ² = 20.568, p = 0.151; χ²/df = 1.371 (< 2.0); comparative fit index (CFI) = 0.999; goodness-of-fit index (GFI) = 0.993; adjusted goodness-of-fit index (AGFI) = 0.975; and normed fit index (NFI) = 0.998. Error indices were also within acceptable limits, with the standardized root mean square residual (SRMR) = 0.015 and root mean square error of approximation (RMSEA) = 0.025—both below the cutoff of 0.05.

Overall, these results demonstrated that the second-order measurement model provided an excellent fit to the data and confirmed the validity and reliability of the SHL-SW construct (Table 4).

## Discussion

This study successfully developed and validated the gender-inclusive SHL-SW, a 10-item instrument with a strong four-factor structure. This section discusses how to understand this structure in the context of prior studies as well as the methodological rigor that supports the scale’s reliability and potential for broader use.

### Step 1: Definition and components of SHL

The 18 multidisciplinary experts comprised policymakers, academic staff, and health care workers. Therefore, the experts provided reliable opinions for the researchers to determine the definition and components of SHL in order to create an SHL-SW questionnaire. In addition, there was a sufficient number of experts, which decreased the error by 0.02 for the Delphi technique [26].

Further, the CFA in this study confirmed that the SHL-SW is best represented by four distinct but interrelated factors: access, understanding, appraisal, and application. This finding is highly significant because it aligns perfectly with the dominant integrated model of health literacy that is widely accepted in international public health research [17,18]. The consistency of our findings with this foundational framework provides strong evidence for the scale’s theoretical soundness and suggests its potential for generalizability beyond the Thai context.

When compared to the local context, a few health literacy models developed for the general Thai population have proposed more extensive frameworks with five to seven components [23,27–29]. However, the four-factor structure of the SHL-SW is a deliberate strength. For a specific, often hard-to-reach population such as sex workers, a concise and targeted instrument is more practical and effective. The four domains identified in this study represent the essential core competencies of health literacy. Crucially, while previous Thai and international studies have focused on the general population or reproductive health, to the best of our knowledge, the SHL-SW is the first instrument specifically developed and validated for the unique needs of gender-diverse sex workers, thus filling a critical gap in the literature [20].

### Step 2: Results of the CVI analysis

The findings from the CVI analysis provide strong evidence of the content validity of the SHL-SW. Initially, most items demonstrated acceptable validity (CVI ≥ 0.83), which is consistent with the minimum acceptable threshold of 0.78–0.80 suggested in previous psychometric literature [30]. However, one item (Item 4) did not meet this criterion in the first round, and several items were judged by experts to lack clarity in wording. These observations highlight the importance of iterative refinement in instrument development, particularly in sensitive contexts, such as SHL among sex workers.

Further, the results of the multi-round Delphi technique provide strong support for the content validity of the SHL-SW. The iterative process of expert consultation was crucial. For example, the initial lower CVI score for one item as well as expert feedback on unclear wording in the first round demonstrated the importance of this refinement process, particularly for a sensitive and nuanced topic such as SHL among sex workers. The subsequent increase in the scale-level CVI (S-CVI) to an excellent 0.94 exceeded the established benchmark of 0.90 [30]. These results demonstrate that the SHL-SW is conceptually sound and linguistically appropriate, thus enhancing its relevance for sex workers of diverse genders in Thailand. Moreover, the rigorous content validation process ensures that the instrument is not only theoretically grounded but also contextually adapted to the sociocultural realities of sex workers in Thailand and other countries. This contributes to the robustness and credibility of the scale as a practical research tool for assessing and promoting sexual health literacy.

### Step 3: Results of the CFA

The proposed four-factor structure of the SHL-SW was strongly supported by the CFA of data from 600 sex workers of various genders. A closer look at the item-level performance revealed a common and significant psychometric problem, even though the second-order model as a whole fit the data very well (RMSEA = 0.025). The two items with the lowest factor loadings were HL5 from the understanding factor and HL7 from the appraisal factor. Their main feature is that they were the only items on the scale with negative wording. Due to variations in cognitive processing, negatively worded items may produce a “method effect,” which makes them correlate less with positively worded items. This phenomenon is well-documented in the literature [31].

Despite this acknowledged difficulty, it is advised to use negatively worded items to reduce acquiescence bias, which is the propensity of respondents to agree with statements regardless of their content [31,32]. The inclusion of such items serves a significant methodological purpose, even though their lower loadings imply that these items contribute less to their respective factors than positive items.

Importantly, the overall model fit was still very good even though these two items performed slightly worse than others. This suggests that the four-factor conceptual model is essentially sound and that the method effect of the negatively worded items was sufficiently insignificant to jeopardize the SHL-SW’s overall construct validity. This finding provides confidence that the scale is a valid and trustworthy measure overall.

A key strength of this study is the development of the SHL-SW, the first gender-inclusive SHL scale specifically for sex workers in Thailand. The instrument’s development was methodologically robust, grounded in a clear conceptual framework, refined by expert consensus via the Delphi technique, and validated with a large, gender-diverse sample (N = 600). This sample size provided sufficient statistical power for rigorous psychometric evaluation, including CFA, thus lending strong support to the scale’s construct validity and reliability. Furthermore, the scale’s brevity (10 items) enhances its feasibility and practical utility for implementation in both research and clinical settings.

A strength that supports the scale’s potential for cross-cultural applicability is the fundamental theoretical alignment of the SHL-SW’s four-factor structure (access, understanding, appraisal, application) with the dominant international health literacy framework [17,18]. This suggests that the core cognitive and social competencies measured by the scale are rooted in universally recognized dimensions of health literacy. Thus, the SHL-SW serve as a foundational measurement model for assessing sexual health literacy among marginalized populations in diverse global settings.

However, the applicability of the scale beyond the Thai context warrants careful consideration. Cross-cultural validation is essential prior to broad implementation. Therefore, future research must prioritize the rigorous cross-cultural validation of the SHL-SW in diverse geographical and socio-cultural settings. We strongly recommend employing measurement invariance testing in future cross-national comparative studies to ensure that the scale measures the same underlying construct consistently across different cultural groups. In practical applications, the SHL-SW could be incorporated into public health programs, clinical settings, and community-based initiatives globally to monitor and enhance SHL among sex workers. Policymakers and practitioners are encouraged to use the instrument as a screening and evaluation tool to design tailored interventions aimed at STD prevention.

## Conclusion

This study successfully developed and validated the gender-inclusive SHL-SW, thus providing the first robust, psychometrically sound instrument tailored to this key population in the Thai context. This 10-item, four-factor scale was established through a rigorous multi-phase process, including expert consensus via the Delphi technique. Its strong construct validity was confirmed by a CFA in a sample of 600 sex workers, which demonstrated an excellent fit to the data (χ² = 20.568, p = 0.151; CFI = 0.99; RMSEA = 0.04).

By filling a critical measurement gap, the SHL-SW provides researchers, practitioners, and policymakers with a valid and reliable instrument for assessing SHL among sex workers. Its application may inform the design of evidence-based interventions and public health strategies aimed at reducing the transmission of STDs and promoting safer sexual health practices.

## Supporting information

S1 Table Sexual health literacy scale for sex workers (SHL-SW). (TIF)

S1 Fig Confirmatory factor analysis (CFA) model of the Sexual Health Literacy Scale for Sex Workers (SHL-SW). (TIF)
